# Relationship Between Urinary Cross-Linked N-Telopeptide of Type-I Collagen and Heel Stiffness Index Measured by Quantitative Ultrasound in Middle-Aged and Elderly Men

**DOI:** 10.1097/MD.0000000000001797

**Published:** 2015-11-06

**Authors:** Takayuki Nishimura, Kazuhiko Arima, Yasuyo Abe, Mitsuo Kanagae, Satoshi Mizukami, Takuhiro Okabe, Yoshihito Tomita, Hisashi Goto, Itsuko Horiguchi, Kiyoshi Aoyagi

**Affiliations:** From the Department of Public Health (TN, KA, YA, TO, YT, KA), Nagasaki University Graduate School of Biomedical Sciences, Nagasaki; Department of Rehabilitation (MK, SM, TO, YT), Nishi-Isahaya Hospital, Isahaya; Goto Health Care Office (HG), Nagasaki; and Center for Public Relations Strategy (IH), Nagasaki University, Nagasaki, Japan.

## Abstract

The aim of the present study was to investigate the age-related patterns and the relationship between levels of urinary cross-linked N-telopeptide of type-I collagen (NTx) and heel stiffness index measured by quantitative ultrasound (QUS) in men with a special reference to age groups of aged 40 to 59 years and ≥60 years.

A total of 379 men participated in this study. Heel stiffness index (bone mass) was measured by QUS. Spot urine samples were collected, and urinary NTx was measured. The values were corrected for creatinine (Cre) concentration.

Stiffness index was significantly lower in men aged ≥60 years compared with men aged 40 to 59 years (*P* < 0.0001). There was no significant difference of Log (NTx/Cre) by 10-year age groups. Multiple regression analysis showed that higher level of urinary NTx/Cre was significantly correlated with lower stiffness index after adjusting for age and body mass index in men aged ≥60 years, but not in men aged 40 to 59 years.

Higher rates of bone resorption were associated with lower stiffness index only in elderly men. Our results may indicate a different mechanism of low bone mass among different age groups.

## INTRODUCTION

Osteoporosis and resulting fractures impair the activities of daily living and quality of life, leading to increased morbidity and mortality in the elderly.^1,2^ Although osteoporosis is more common in women, men have substantial age-related decreases in bone mineral density (BMD).^3–5^ Thus, osteoporosis in men is also a significant public health problem because of the rapid aging of society.

Cross-linked N-telopeptide of type-I collagen (NTx) is one of the biochemical markers of bone resorption, and is widely used in clinical situations to evaluate the indication and efficacy of treatments for osteoporosis.^6–8^ Previous studies showed that the serum or urinary concentration of NTx increased after menopause in women.^5,9–11^ From 40 years on, age-related patterns of NTx in men have been controversial. Some studies reported that NTx increased with age,^10,12^ but the others reported that NTx was stable.^5,13–15^

Several studies reported that NTx correlated inversely and significantly with BMD at some skeletal sites in men,^10,13^ but these correlations were weaker than those in women.^10^ On the contrary, data on relationship between bone turnover marker and bone quantitative ultrasound (QUS) measurement are limited, especially in men. QUS is a developed promising technique for evaluation of fracture risk and bone mineral status. In addition to the features of portability, relatively low cost, and ease to use, QUS is also free from ionizing radiation and provides information on bone structure.^16^ Therefore, QUS has been widely used in Japan for screening patients at high risk of osteoporotic fractures.^17,18^ Recently, Boonen et al^19^ showed that higher levels of bone markers serum N-terminal propeptide of type 1 procollagen and β-isomerized C-terminal telopeptides were significantly associated with lower QUS parameters among men aged 40 to 79 years; however, the study was conducted among combining middle-aged and elderly men. Thus, the relationship between NTx and bone mass (QUS) in men is still unclear, and there were few studies with references to age groups.^20^

The aim of the present study was to investigate the age-related patterns and the relationship between levels of urinary NTx and heel stiffness index measured by QUS in men with a special reference to age groups (40–59 years, and ≥60 years).

## METHODS

The subjects were community-dwelling men aged ≥40 years at Nagasaki Prefecture, Japan, who were invited to participate in periodic health examinations in 2006 to 2009. A total of 379 men participated in this study. All subjects gave written informed consent before examination. This study was approved by the Institutional Review Board of Nagasaki University.

Heel stiffness index (bone mass) by QUS was measured using a Lunar Achilles device (GE Lunar Corp., Madison, WI). Spot urine samples (8:00–10:00 am) were collected. Urinary NTx, a marker of bone resorption, was measured with enzyme immunoassay. The values were corrected for creatinine (Cre) concentration. Height (m) and weight (kg) were measured with light clothing and without shoes, and body mass index (BMI) was calculated as weight (kg)/height^2^ (m^2^).

### Statistical Analysis

The data were analyzed using the Statistical Analysis System software package version 9.2 (SAS Institute, Cary, NC). Because NTx/Cre was not normally distributed, NTx/Cre was treated as Log (NTx/Cre). Student *t* test was used to examine the difference in variables between the age groups. One-way ANOVA was used to Log (NTx/Cre) between 10-year age groups. Simple correlation analysis was used to examine the correlation between the levels of NTx/Cre and stiffness index. Multiple linear regression analysis was used to explore the effect of age, BMI and Log (NTx/Cre) on stiffness index. A *P* value of <0.05 was considered as statistically significant.

## RESULTS

Table 1 shows the characteristics of our 379 subjects. Weight and height were significantly smaller in men aged ≥60 years compared with men aged 40 to 59 years (*P* < 0.0001). Stiffness index was significantly lower in men aged ≥60 years compared with men aged 40 to 59 years (*P* < 0.0001). There was no significant difference of BMI and Log (NTx/Cre) between age groups.

**TABLE 1:**
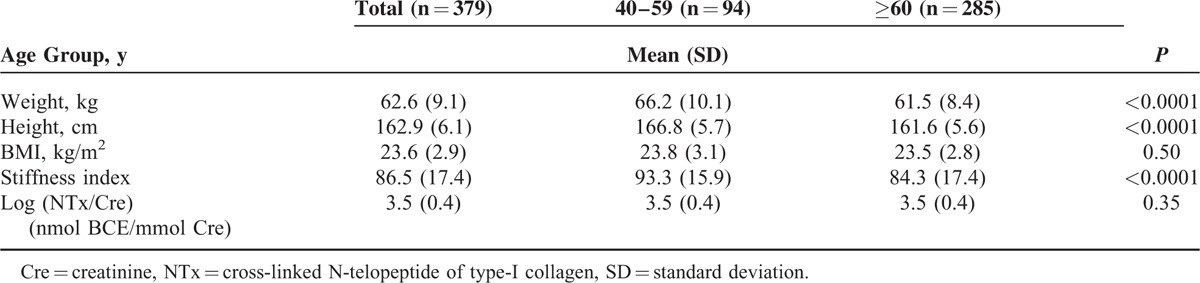
Characteristics of the Study Subjects (n = 379)

In the one-way ANOVA, there was no significant difference of Log (NTx/Cre) by 10-year age groups (*P* = 0.12) (Table 2).

**TABLE 2:**
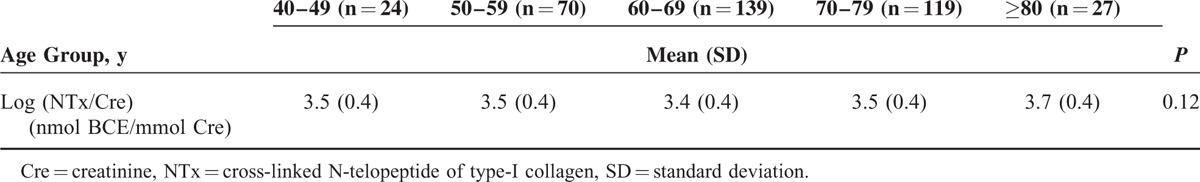
Mean (SD) of Log (NTx/Cre) by Age Group

**FIGURE 1:**
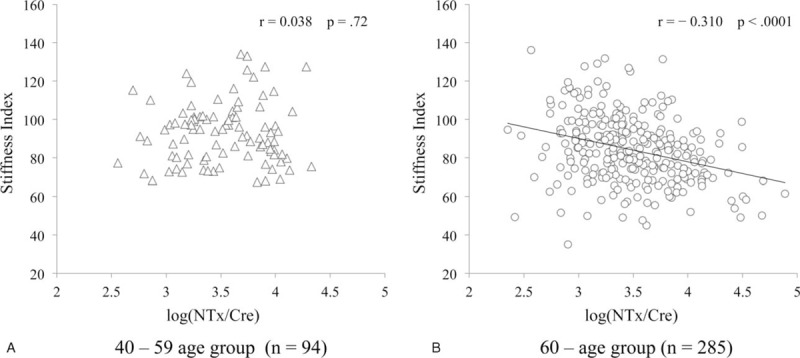
(A) Correlation between Log (NTx/Cre) and stiffness index in 40 to 59 age group (*r* = 0.038, *P* = 0.72). (B) Correlation between Log (NTx/Cre) and stiffness index in ≥60 age group (*r* = −0.310, *P* < 0.0001).

In men aged over 60 years, there was a significant negative correlation between the levels of urinary NTx/Cre and stiffness index, but not in men aged 40 to 59 years (Figure 1).

In order to assess further the independent relationship, we used a multiple regression analysis. It showed that higher level of urinary NTx/Cre was significantly correlated with lower stiffness index (QUS) after adjusting for age and BMI in men aged ≥60 years, but not in men aged 40 to 59 years (Table 3). In addition, NTx/Cre was also significantly correlated with lower stiffness index (QUS) after adjusting for age and height/weight in men aged ≥60 years (data not shown).

**TABLE 3:**
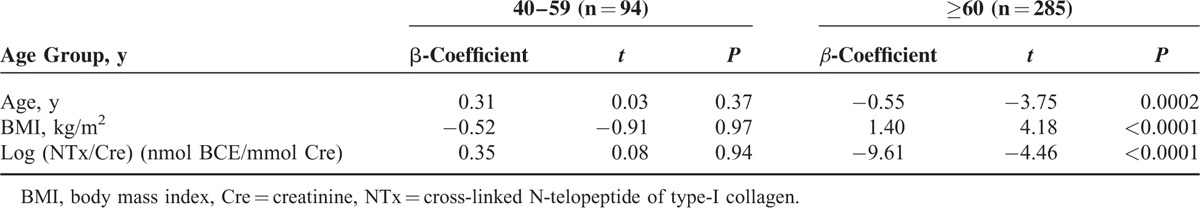
Multiple Linear Regression Analysis of Stiffness Index

## DISUCUSSION

We showed that higher level of urinary NTx was significantly correlated with lower stiffness index (QUS) after adjusting for age and BMI in men aged ≥60 years, but not in men aged 40 to 59 years. Boonen et al^19^ reported that higher levels of bone markers were significantly associated with lower QUS parameters among middle-aged and elderly men combined, but did not analyze with a special reference to middle-aged or elderly men. To the best of our knowledge, we first reported the significant correlation between NTx and stiffness index (QUS) only in elderly men, not in middle-aged men.

We showed no significant differences of urinary NTx among 10-year age groups, which is consistent with previous reports in men aged ≥40 years.^5,14,15^ In women, NTx values increase with the decline in estrogen production at the time of menopause and remain elevated thereafter.^21^ Because men do not have the equivalent of menopause, NTx may not increase rapidly in men. However, some studies reported increasing of NTx with age in middle-aged and elderly men.^10,12^ Further study is needed to explore the age-related patterns of NTx in middle-aged and elderly men.

Our results showed significant and negative correlation between NTx and stiffness index (QUS) only in men aged ≥60 years. Khosla et al^10^ showed significant and negative association between proximal femur BMD and serum NTx in men aged ≥50 years, but not in men <50 years. Chandani et al^13^ showed that higher serum NTx correlated with lower femoral neck BMD in men aged 68 to 89 years. These findings suggest that higher rates of bone resorption are associated with lower bone mass, especially in elderly men.

In elderly men, slightly increased bone resorption is not matched by a parallel increase in bone formation, and this imbalance results in the age-related bone loss.^20^ Unfortunately, bone formation marker was not measured in our study. It is needed to evaluate both resorption and formation markers to understand more detailed mechanisms of osteoporosis in elderly men.

Our study has several limitations. First, because this study was cross-sectional design, our results do not necessarily show a causal relationship. Second, information on other determinants (eg, genetic background, nutritional status, socioeconomic status, medication [ex. corticosteroids], levels of Vitamin D, and underlying comorbidities) contributing to skeletal maintenance in aging men^19^ was not available to our study. Finally, the subjects were participants of health examination and may not be representative of the general population.

In conclusion, we showed that higher level of urinary NTx was significantly correlated with lower stiffness index (QUS) in men aged ≥60 years, but not in men aged 40 to 59 years. Higher rates of bone resorption were associated with lower stiffness index only in elderly men. Our results may indicate different mechanisms of low stiffness index among different age groups.
